# Alterations of gut microbiota in Down syndrome and their association with Alzheimer's disease

**DOI:** 10.1002/alz.71815

**Published:** 2026-09-08

**Authors:** Camilla Pellegrini, Francesco Ravaioli, Sara De Fanti, Alessia Siliquini, Claudia Sala, Magali Rochat, Virginia Pollarini, Barbara Polischi, Alberto Pasti, Margherita Grasso, Maria Rambaldi, Francesco Cardoni, Nicola Grotteschi, Filippo Caraci, Pietro Cortelli, Federica Provini, Raffaele Lodi, Luca Morandi, Piero Parchi, Gian Luca Pirazzoli, Luisa Sambati, Caterina Tonon, Maria Giulia Bacalini

**Affiliations:** ^1^ IRCCS Istituto delle Scienze Neurologiche di Bologna Bologna Italy; ^2^ Department of Medical and Surgical Sciences (DIMEC) University of Bologna Bologna Italy; ^3^ Oasi Research Institute ‐ IRCCS Troina Italy; ^4^ Department of Biomedical and Neuromotor Sciences (DIBINEM) University of Bologna Bologna Italy; ^5^ Department of Drug and Health Sciences University of Catania Catania Italy

**Keywords:** Alzheimer's disease, Down syndrome, gut microbiota, major neurocognitive disorder, metagenomics, plasma biomarkers

## Abstract

**INTRODUCTION:**

Adults with Down syndrome (DS) have a higher risk of Alzheimer's disease (AD). As gut microbiota (GM) alterations have been reported in AD, we investigated their association with cognitive decline and plasma AD biomarkers in DS.

**METHODS:**

Fecal and plasma samples were collected from 58 adults with DS (21–75 years) and 30 euploid controls (CTRL; 25–83 years). GM was profiled using 16S rRNA sequencing, filtering low prevalent taxa. Major neurocognitive disorder (NcD) was diagnosed with Diagnostic and Statistical Manual of Mental Disorders, Fifth Edition (DSM‐5) criteria. Plasma levels of phosphorylated tau 181 (p‐tau181), neurofilament light chain (NfL), and glial fibrillary acidic protein (GFAP) were measured using Simoa.

**RESULTS:**

DS showed no changes in overall microbial diversity compared to CTRL, but genera including UBA1819 and *Intestinibacter* were altered. Specific genera showed changes in DS with NcD, like *Alistipes* (increased) and *Roseburia* (decreased), with the latter negatively associated with plasma AD biomarkers.

**DISCUSSION:**

Adults with DS display AD‐associated changes in GM partially resembling those reported previously in euploid AD patients.

## Highlights

- Gut microbiota (GM) was evaluated in a cohort of adults with Down syndrome (DS) and euploid controls
- Taxa altered in DS have been reported to be associated with mild cognitive impairment
- *Roseburia* decreased in DS with major neurocognitive disorder, similarly to patients with Alzheimer's disease (AD)
- *Roseburia* genus was also associated with plasma AD biomarkers in DS

## 1 BACKGROUND

Down syndrome (DS) is a genetic disorder caused by complete or partial triplication of chromosome 21. DS occurs in ≈1–2 per 1000 live births globally, with a worldwide prevalence of 1 in 800.^1^ Individuals with DS present an increased risk for various health issues, including congenital heart disease, developmental delay, leukemia, obesity, obstructive sleep apnea, and abnormalities in myelopoiesis and inflammatory responses.^2^, ^3^, ^4^ Alterations in brain development lead to a reduction in total brain volume, particularly in the cortical, hippocampal, and cerebellar regions, and are associated with intellectual disability (ID) of variable severity.^5^ Furthermore, the overexpression of the amyloid precursor protein (*APP*) gene, located on chromosome 21, increases the risk of developing Alzheimer's disease (AD)–related dementia in adults with DS.^6^ By age 40, pathological features of AD are already detectable in cerebrospinal fluid (CSF) and plasma, as demonstrated by several studies reporting elevated levels of neurofilament light chain (NfL), phosphorylated tau (p‐tau181, p‐tau217), glial fibrillary acidic protein (GFAP), and a decline in the ratio of proteins amyloid beta (Aβ)42/40.^7^, ^8^, ^9^, ^10^ Premorbid ID level does not affect the timing of AD onset and progression in DS,^11^ whereas individuals with DS who carry the apolipoprotein E (*APOE*) ε4 allele show earlier clinical and AD biomarker changes, similarly to what is observed in the euploid population.^12^ Besides the *APOE* genotype, the age at onset and progression of dementia in DS are highly variable, suggesting the existence of factors that can modulate the AD pathological cascade, as observed in the general population.

The gut microbiome represents the most densely populated bacterial community in the human body and plays an essential role in maintaining overall health. Intestinal bacteria and their metabolites support key functions including preserving gut barrier integrity, modulating the immune system, and regulating essential metabolic processes.^13^ Moreover, the gut microbiome can influence brain function and homeostasis through the bidirectional communication network known as the gut–brain axis. This axis transmits information via neural, immune, and endocrine pathways, thereby impacting mood and cognitive functions, including memory, cognition and social behavior, and its alterations can contribute to the onset and progression of neurological and psychiatric disorders.^14^, ^15^, ^16^, ^17^, ^18^ Several studies have suggested a link between alterations in the gut microbiome taxa composition (referred as gut microbiota [GM]), AD, and its preclinical conditions.^19^, ^20^, ^21^, ^22^, ^23^ Altered GM may contribute to AD pathogenesis through several mechanistic pathways. Dysbiosis has been linked to elevated production of lipopolysaccharides (LPS) and microbial amyloids, which can compromise intestinal epithelial integrity and disrupt the blood–brain barrier (BBB). These alterations facilitate systemic and neuroinflammatory responses, promote oxidative stress, sustain amyloid beta (Aβ) aggregation, induce central insulin resistance, and ultimately lead to neuronal apoptosis, all of which are characteristic features of AD neuropathology.^24^, ^25^, ^26^

Studies investigating the gut microbiome in individuals with DS are limited, but available results suggest an imbalance in both GM and metabolite profile compared to euploid subjects. These differences were evident even during childhood and have been associated with cognitive and behavioral disturbances.^27^, ^28^, ^29^ Of interest, a recent metagenomic study investigated GM in a cohort of 20 DS individuals that were cognitively stable or with mild cognitive impairment (MCI) and reported both overall and taxa‐specific changes according to cognitive status.^30^

The present study seeks to advance understanding of the role of GM in individuals with DS, with particular focus on its association with the development of AD. We investigated the fecal microbiota of adults with DS using 16S ribosomal RNA (rRNA) high‐throughput sequencing and analyzed its association with plasma levels of AD‐related biomarkers,^31^ including phosphorylated p‐tau181, NfL, and GFAP.

## 2 METHODS

### 2.1 Cohort

Participants with DS and healthy controls were recruited at Azienda Unità Sanitaria Locale (AUSL) Bologna—IRCCS Istituto delle Scienze Neurologiche of Bologna (Italy) in the framework of the study protocols “Study of aging in people with Trisomy 21: longitudinal evaluation of molecular markers and clinical‐functional, neuropsychological and cognitive aspects (AgingInT21)” and “Digital biomarkers in Parkinson and Alzheimer diseases and in subjects with Down syndrome (DARE‐T21).”^32^ The protocols were approved by the local ethics committee of the local health service of Bologna, Italy (1070‐2021‐SUPER‐AUSLBO and 378‐2024‐OSS‐AULSBO) and written informed consent was obtained from the participants and from their relatives or legally authorized representatives. Geriatric and neurological evaluation was performed by expert clinicians according to a standardized protocol, as described previously.^33^, ^34^ Dementia was defined as an objective and progressive decline in cognitive and functional performance based on Diagnostic and Statistical Manual of Mental Disorders, Fifth Edition (DSM‐5) criteria and, according to DSM‐5 terminology, is referred to as major neurocognitive disorder (NcD) in the text.^35^ None of the participants, either DS or euploid subjects (CTRL), had received antibiotic therapy in the month preceding sample collection.

### 2.2 Sample collection and DNA extraction

Fecal samples were collected in OMNIgene•GUT tubes to stabilize microbial DNA (DNA Genotek, Canada). Samples were stored for a maximum of 7 days at room temperature, vortexed 60 s, transferred to cryotubes, and stored at –80°C. Microbial DNA was extracted from feces using a modified version of the protocol described by Ghosh et al.^36^ Briefly, 200 𝜇L of defrosted faeces were lysed using the ASL buffer (QIAGEN, Hilden, Germany) and bead beaten 2 times at 1800 rpm for 3 min, with a 1‐min pause between the two cycles, in Power Bead Tubes (ceramic 1.4 mm) using the PowerLyzer 24 Homogenizer (QIAGEN, Hilden, Germany). After proteinase K treatment, DNA purification was performed using the QIAamp Fast DNA Stool Mini Kit (QIAGEN, Hilden, Germany). DNA was quantified using Qubit dsDNA Broad Range (BR) Assay Kit (QIAGEN, Hilden, Germany) and stored at –20°C before analysis.

**RESEARCH IN CONTEXT**

1. **Systematic review**: A comprehensive review of the existing literature was carried out to investigate the role of gut microbiota (GM) in individuals with Down syndrome (DS) in the context of Alzheimer's disease (AD) progression. Research on GM composition in DS remains limited, and only a few studies have examined the relationship between GM, neurocognitive decline, and plasma AD biomarkers in DS.
2. **Interpretation**: Although the overall GM composition was similar between DS and euploid controls, we observed changes at specific taxa reported previously in patients with mild cognitive impairment. Furthermore, we found alterations in the GM of DS with major neurocognitive disorder and in association with AD plasma levels, that resemble those reported previously in euploid patients with AD.
3. **Future directions**: Future research should validate these findings in larger cohorts and integrate additional characterizations, such as fecal metabolomic profiling, to better elucidate the contribution of GM to the pathogenesis and progression of AD in adults with DS.

### 2.3 Analysis of plasma AD biomarkers

Venous blood was collected at fasting using sodium ethylenediaminetetraacetic acid (EDTA) tubes. Blood samples were centrifuged at 2000 × *g* at room temperature for 10 min within 2 h from collection. Plasma supernatant was collected, divided into aliquots, and frozen at −80°C until use. Plasma p‐tau181, NfL, and GFAP were measured with SiMOA p‐tau181 advantage V2 and SiMOA Neurology 2‐Plex B (GFAP, NF‐l) Assay Kit, respectively. Analyzes were performed on a SiMOA SR‐X analyzer platform (Quanterix, Billerica, MA, USA). Biomarker levels were transformed using a base‐2 logarithm.

### 2.4 *APOE* genotyping

Blood samples were collected in EDTA tubes and stored at –20°C. Genomic DNA was extracted from 200 µL of whole blood using the QIAamp DNA Blood Mini Kit (QIAGEN, Hilden, Germany), according to the manufacturer's instructions. DNA was quantified using Qubit dsDNA Broad Range (BR) Assay Kit (QIAGEN) and stored at –20°C before analysis. Apolipoprotein E (*APOE*) genotyping was performed by real‐time using TaqMan Fast Advanced Master Mix and TaqMan probes (Thermo Fisher Scientific, Waltham, MA) for rs7412 (C__904973_10) and rs429358 (C__3084793_20). Polymerase chain reaction (PCR) amplification was conducted on CFX Opus 96 Real‐Time PCR System (Bio‐Rad Laboratories, Hercules, CA, USA).

### 2.5 Library construction and sequencing

Library preparation was conducted following Illumina 16S Library Preparation Workflow. Specifically, the 16S rRNA gene hypervariable V3–V4 region was amplified with primers 341F and 805R. The V3–V4 region is generally chosen for GM studies because it offers a proven, highly cited, and cost‐effective method that yields high taxonomic resolution.^37^ The PCR products were purified using MagSi Prep Plus magnetic beads (Magtivio, Nuth, The Netherlands) and amplified for eight cycles using Nextera XT Indexed Primers. The final amplicons were purified, quantified using Qubit dsDNA BR Assay Kit) and normalized at equimolar concentration (4 nM). Pooled libraries were denatured with 0.2 N NaOH, diluted, and combined with 10% PhiX Sequencing control V3. The final pool (6 pM) was sequenced with MiSeq v3 reagents (Illumina, San Diego, CA) on Illumina MiSeq platform using paired‐end 2 × 300‐bp reads.

### 2.6 Bioinformatic processing

Pre‐processing of raw paired‐end reads was performed according to Dada2 Big Data pipeline^38^ in R (v. 4.2.2). Briefly, forward and reverse reads were checked for quality and trimmed to remove Illumina adapters and low‐quality sequences. Filtered reads were deduplicated, merged, and clustered into an amplicon sequence variant (ASV) table. Finally, after removal of chimeric ASVs, taxonomy was assigned against SILVA non‐redundant small subunit rRNA database (v138.1),^39^ with default bootstrap confidence value (50%). Samples with low sequencing depth (<10,000 reads) were excluded from the analysis (88 samples remaining). Taxa that were assigned to neither Bacteria nor Archaea and taxa present in less than 10% of the total sample size were removed from the ASV count table.

### 2.7 Statistical analyses

All statistical analyzes were performed with R (v. 4.2.2). Age and sex were included in all parametric statistical models as confounding variables. For analyses performed within the DS group, body mass index (BMI) was included as a covariate (correction was not applied to euploid controls due to unavailability of BMI data). Statistically significant threshold was considered at nominal *p‐*values < 0.05; in addition, false discovery rate (FDR) according to the Benjamini–Hochberg (BH) method was calculated. Alpha and beta diversity at ASVs level were calculated using the *phyloseq* package (v 1.42.0).^40^ Alpha diversity was calculated according to the number of observed species, Chao, Shannon, and inverted Simpsons indexes. Wilcoxon rank‐sum test was used to compare alpha diversity measurements between that DS and CTRL groups, as alpha diversity data was not normal according to the Shapiro–Wilk test. The association between alpha diversity metrics and levels of plasma markers p‐tau181, NfL, and GFAP in DS subjects was evaluated by fitting a linear regression model. Beta diversity was assessed via principal component analysis (PCoA). Distance matrices were computed using Bray–Curtis dissimilarity, which quantifies differences in taxa abundances between samples; unweighted UniFrac, which measures phylogenetic dissimilarity based on the presence or absence of lineages; and weighted UniFrac, which incorporates taxonomic relatedness through phylogenetic branch lengths, with taxa relative abundances, thereby capturing both evolutionary relationships and their dominance within each community. In addition, for each diversity metric, beta dispersion was computed using the *PERMDISP* R package. PERMANOVA function in *vegan* R package (v 2.6‐6.1) was used to compare beta‐diversity metrics and beta‐dispersion distances between DS and CTRL participants, as well as between DS with and without NcD. Alterations in microbial abundances were evaluated at the phylum, family, and genus level. First, dysbiosis was evaluated by calculating Firmicutes/Bacteroidetes ratio and compared between DS and CTRL participants using ANOVA. Second, DESeq2 pipeline^41^ using Wald test was used to perform differential microbial abundance analyzes between DS and CTRL, as well as between DS with and without NcD. An alternative analysis of differential abundance was performed using the Aldex2 method.^42^ The association between microbial abundance and AD plasma biomarkers was also assessed using the DESeq2 pipeline. Finally, predictive functional profiling was performed using PICRUSt2 (v. 2.6.2).^43^ Predicted abundances were normalized with centered log‐ratio (CLR) transformation using the compositions package (v. 2.0‐6). Differential pathway abundance analysis in DS versus CTRL and in DS with NcD versus DS without, as well as the association of pathway abundance with AD biomarkers, were conducted in R using *limma* package (v. 3.54.2).

## 3 RESULTS

### 3.1 Description of the cohort

After quality filtering, metagenomic data were available for 58 adults with DS(20 female, mean age 42 years) and 30 euploid controls (CTRL; 24 female, mean age 58 years). Mean value of BMI in DS was 27.7 (13 with BMI >30, obese). BMI values were not available for CTRL and for four participants with DS. Four DS participants had chronic gastrointestinal diseases, including gluten intolerance (*n =* 1), chronic diarrhea (*n =* 1), dyspepsia and celiac disease (*n =* 1), and congenital gastrointestinal malformations (*n =* 1).

Of the 58 participants with DS, 10 had the ε2/ε3 genotype, 38 the ε3/ε3 genotype, and 10 the ε3/ε4 genotype, whereas genotype data were not available for CTRL. Nine DS participants had a diagnosis of major neurocognitive disorder (or NcD), of which two were ε4 allele carriers. Demographic and clinical characteristics of the cohort are summarized in Table 1, while *APOE* genotype distribution is reported in Table 2.

**TABLE 1:** Demographic characteristics of participants.

	CTRL group (*n =* 30)	DS group (*n =* 58)	*p*
*Demographic*			
Age, years, mean (range)	58 (25–83)	42 (21–75)	<0.001^§^,^*^
Female, *n*/total	24/30	20/58	<0.001**^†^** **^,^** ^*^
BMI, mean ± SD	/	27.7 ± 5.9	/
*Dementia clinical diagnosis*			
Major neurocognitive disorder, *n*	/	9	/
*Comorbidities n*			
Gastrointestinal disorders	/	4	/
Type 2 diabetes	/	0	/

Abbreviations: BMI, body mass index; CTRL, euploid subjects; DS, Down syndrome; *n*, number; *p*, *p*‐value; SD, standard deviation.

^*^ Significant.

^†^ Calculated by chi‐square (χ2) test.

^§^ Calculated by *t*‐test.

**TABLE 2:** Demographic characteristics of DS groups and relative subgroups based on NcD diagnosis.

Variable	DS (*n =* 58)	DSw/oNcD (*n =* 49)	DSwNcD (*n =* 9)	*p*
**Demographic**				
Age, years, mean (range)	42 (21–75)	38 (21–60)	58 (48–75)	<0.001^*^, ^§^
Female, *n*/total	20/58	16/49	4/9	0.76^†^
BMI, mean ± SD	27.7 ± 5.9	27.8 ± 6.1	26.8 ± 4.7	0.66^§^
*APOE* ɛ4 allele carrier, *n* (%)	10 (17%)	8 (16%)	2 (22%)	1^†^

Abbreviations: *APOE*, apolipoprotein E; BMI, body mass index; DS, Down syndrome; *DSwNcD*, Down syndrome with major neurocognitive disorder; *DSw/oNcD*, Down syndrome without major neurocognitive disorder; *n*, number; *p*, *p*‐value; SD, standard deviation.

^*^ Significant.

^†^ Calculated by chi‐square (χ2) test.

^§^ Calculated by *t*‐test.

Medication use and polypharmacy tend to be highly prevalent among adults with DS.^44^ In our cohort, information on medications and nutritional supplements were available only for participants with DS and are detailed in Table S1. The use of anxiolytic and antipsychotic medications was significantly higher in DS with NcD (Table S1).

### 3.2 Plasma AD biomarkers

**FIGURE 1:**
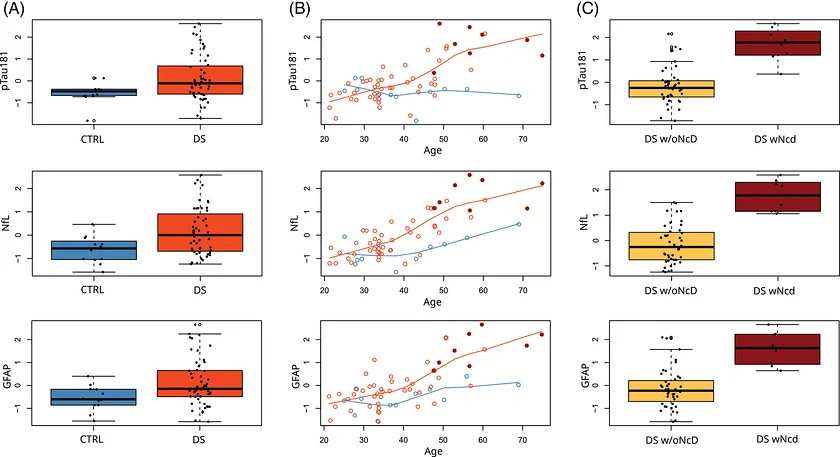
Plasma levels of p‐tau181, NfL, and GFAP. A. Boxplots of p‐tau181, NfL, and GFAP levels in DS (red) and CTRL (blue) groups. Sample size *n =* 63. B. Scatterplots of p‐tau181, NfL, and GFAP levels with respect to the age of DS (red) and CTRL (blue); DS with NcD are colored in dark red. Sample size *n =* 63. C. Boxplots of p‐tau181, NfL, and GFAP levels in DS without NcD (DS w/oNcD, yellow) and DS with NcD (DS wNcD, dark red). Plasma biomarker levels are reported as z‐scores of base‐2 logarithm‐transformed values. Sample size *n =* 51. Abbreviations: CTRL, euploid subjects; DS, Down syndrome; GFAP, glial fibrillary protein; NcD, major neurocognitive disorder; NfL, neurofilament light chain; p‐tau181, phosphorylated tau 181; DS w/oNcD, Down syndrome without major neurocognitive disorder; DS wNcD, Down syndrome with major neurocognitive disorder.

Plasma levels of AD biomarkers (p‐tau181, NfL, and GFAP) were available for 51 DS (19 female; mean age 40 years; 9 with a diagnosis of NcD) and 12 CTRL (8 female; mean age 40 years). As expected,^33^ biomarker levels were higher in DS compared to CTRL (ANOVA correcting for age and sex; p‐tau181: *p‐*value = 0.018; NfL: *p‐*value < 0.001; GFAP: *p‐*value = 0.001; Figure 1A) and showed a relevant increase after 40 years in the DS group (Figure 1B). Furthermore, p‐tau181 and NfL were significantly higher in DS with NcD than in DS without NcD (ANOVA correcting for age and sex; p‐tau181: *p‐*value = 0.025; NfL: *p‐*value = 0.017; Figure 1C).

### 3.3 Analysis of GM in DS and CTRL groups

The 16S rRNA amplicon sequencing yielded a total of 2,284,703 reads (ranging from 11,177 to 57,267 per sample) passing quality filters. After prevalence filtering, the dataset comprised 275 ASVs that were assigned to 6 phyla, 21 families, and 62 genera.

**FIGURE 2:**
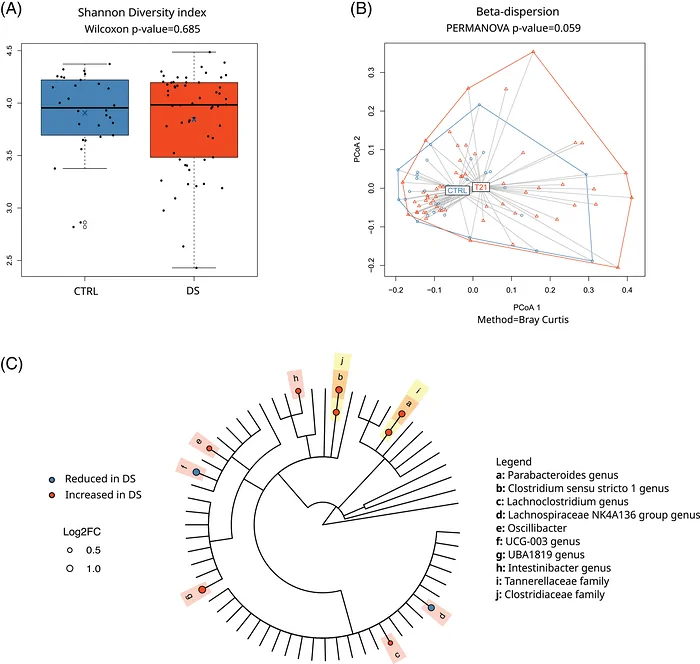
Microbial diversity and taxa abundance differences between the DS and CTRL groups. A. Boxplot showing alpha‐diversity values calculated according to Shannon index in DS and CTRL groups. Group means are indicated with a blue X symbol; group medians are indicated with a continuous black line. Sample size *n =* 88. B. PERMDISP beta‐dispersionPCoA plot showing separation of DS (red) and CTRL (blue) groups based on Bray–Curtis distances. Sample size *n =* 88. C. Cladogram showing different abundant taxa levels in the DS group as identified by Deseq2 analysis. Microbial families showing significant changes are highlighted in yellow, whereas genera are highlighted in orange. Blue and red dots indicate taxa significantly reduced and increased in the DS group, respectively. The dimension of each dot is proportional to the Log2 Fold Change (Log2FC). Sample size *n =* 88.

As a first step, we evaluated differences between CTRL and DS groups in terms of overall microbiota diversity (alpha and beta diversity) and microbial abundance at the phylum, family, and genus levels.Alpha diversity, which is a within‐sample measure of microbiota diversity, did not show significant differences between DS and CTRL groups (Wilcoxon test *p‐*value > 0.05; Figure 2A and File S1). The analysis of beta diversity, which measures microbial community dissimilarities among groups, showed significant differences between DS and CTRL groups with Bray–Curtis distance (*p‐*value = 0.020; *R^2 ^=* 0.020), but not with Weighted and Unweighted Unifrac metrics (File S1). PCoA with unweighted Unifrac metric (Figure S1A) revealed a clear separation of samples on PC1, which was not ascribable to any demographic, clinical, or technical variable in our dataset. Beta‐dispersion analysis, which measures the compositional variation of the microbiome among a group of individuals, returned marginally significant results when using Bray–Curtis distances (*p‐*value = 0.059, *R^2 ^=* 0.034) (Figure 2B; Figure S1A and File S1 ).

We used DESeq2 to analyze differential microbial abundance in DS compared to CTRL participants at different taxonomic levels (phylum, family, and genus) (File S1).

The analysis of bacterial phyla abundance revealed no statistically significant differences between groups. Furthermore, the ratio of Firmicutes to Bacteroidetes (F/B ratio) was not altered in DS compared to CTRL (Figure 2C and File S1). At the family level, we found that the abundance of *Tennerellaceae* and *Clostridiaceae* was significantly higher in DS than in CTRL (nominal *p‐*value = 0.012, 0.012, respectively; Figure 2C and File S1). Finally, we identified eight genera that showed significant abundance alterations in DS compared to CTRL Figure 2C and File S1): *UCG‐003* (nominal *p‐*value = 0.039) and *Lachnospiraceae NK4A136 group* (nominal *p‐*value = 0.002) were reduced in DS, whereas *Lachnoclostridium* (nominal *p‐*value = 0.044)*, Oscillibacter* (nominal *p‐*value = 0.021)*, Intestinibacter* (nominal *p‐*value = 0.020)*, Parabacteroides* (nominal *p‐*value = 0.025)*, Clostridium sensu stricto 1* (nominal *p‐*value = 0.004), and *UBA1819* (nominal *p‐*value < 0.001) genera were increased in DS. For *UBA1819*, statistical significance survived also after FDR correction (*q‐*value = 0.028) (Figure 2C and File S1).

To confirm the above‐described results, we used an alternative analytical approach based on *Aldex2* package. This analysis confirmed the alterations between DS and CTRL for *Parabacteroides* *, Intestinibacter*, and *Lachnospiraceae NK4A136 group* genera (nominal *p‐*value = 0.029, 0.020, and 0.043, respectively). Moreover *Lachnospiraceae UCG‐010, Clostridium sensu stricto 1*, *and UBA1819* were marginally significant (nominal *p‐*value = 0.056, 0.059, and 0.059, respectively) (File S2).

Finally, functional abundance analysis performed using PICRUSt2 tool identified 32 significant pathways in the comparison between DS and CTRL groups (File S3). Considering the higher‐level category annotations, we found that DS showed a reduction in several significant pathways related to quinol and quinone biosynthesis (12/32), including menaquinone (vitamin K2) biosynthesis.

### 3.4 GM analysis according to clinical diagnosis of NcD

To evaluate a possible association between GM composition and cognitive status in adults with DS, we compared DS subjects with and without a clinical diagnosis of NcD. Characteristics of DS with and without NcD are reported in Table 2.

**FIGURE 3:**
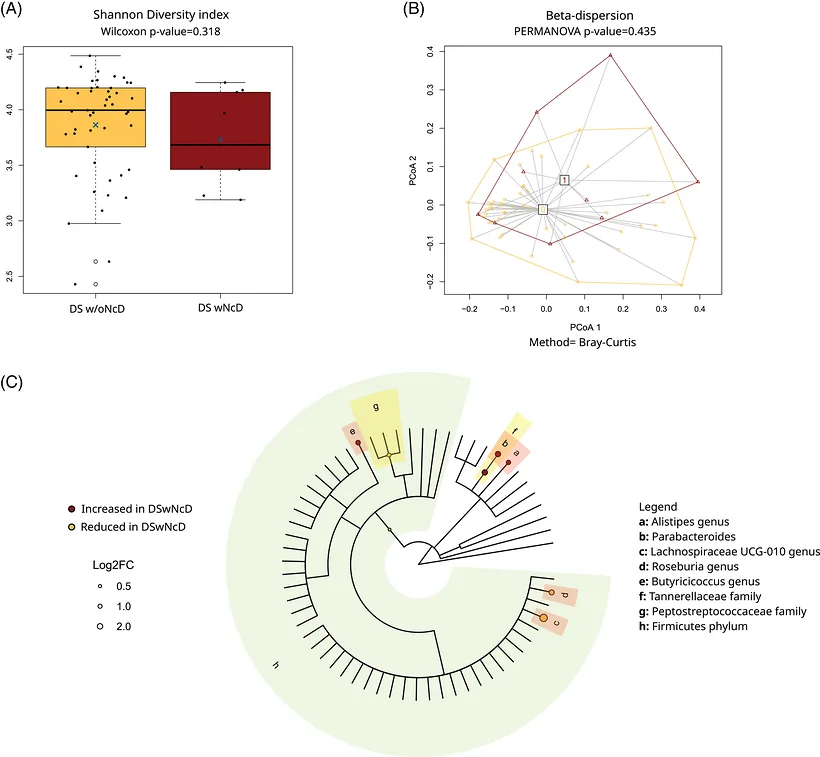
Microbial diversity and taxa abundance differences between DS with and without NcD. A. Boxplot showing alpha‐diversity values calculated according to Shannon index in DS wNcD and w/oNcD groups. Group means are indicated with a blue X symbol; group medians are indicated with a continuous black line. Sample size *n =* 58. B. PERMDISP beta‐dispersion PCoA plot showing the distribution of DS wNcD (dark red) and DS w/oNcD (yellow) groups based on Bray–Curtis distances. Sample size *n =* 58. C. Cladogram showing the differentially abundant taxa levels between DS wNcD and w/oNcD groups as identified by DeSeq2 analysis. Microbial phyla showing significant changes are highlighted in green, families in yellow, and genera are highlighted in orange. Taxa significantly increased in the DS wNcD group are indicated with dark red dots, whereas those reduced in DS wNcD are indicated in yellow. The dimension of each dot is proportional to the Log2 Fold Change (Log2FC). Sample size *n =* 58.

Analysis of alpha diversity did not reveal significant differences for any index (Figure 3A). Only beta diversity calculated with unweighted UniFrac index differed between DS with and without NcD (*p‐*value = 0.047; *R^2 ^=* 0.039) but, as described, this result should be interpreted with caution because PCA according this metric showed a clear separation of samples on PC1 not ascribable to biological or technical reasons Figure S1B and File S4). Beta‐dispersion analysis did not show significant differences between the two groups (Figure 3B; Figure S1B and File S4).

In the analysis of taxa abundance, the *Firmicutes* phylum abundance was significantly reduced in DS with NcD (nominal *p‐*value = 0.048), while no significant differences emerged in the F/B ratio (data not shown). At the family level, DS with NcD showed a reduction of *Peptostreptococcaceae* (nominal *p‐*value = 0.023) and an increase of *Tannerellaceae* (nominal *p‐*value = 0.027) (Figure 3C and File S4). At the genus level, DS with NcD showed a decrease of *Roseburia* (nominal *p‐*value = 0.004) and *Lachnospiraceae UCG‐010* (nominal *p‐*value = 0.005), and an increase of *Parabacteroides* (nominal *p*‐value = 0.017), *Alistipes* (nominal *p‐*value = 0.037), and *Butyricicoccus* (nominal *p*‐value = 0.039) (Figure 3C and File S4).

To account for the possible confounding effects of medications on microbiota composition, we repeated the analysis of taxa abundance including as covariates the drug categories that were significantly more prevalent in DS with NcD (i.e., anxiolytics and antipsychotics). The differentially abundant taxa described largely survived or were marginally statistically significant when anxiolytics or antipsychotics were included as covariates (Table S2).

Finally, functional abundance analysis identified two significant pathways (Biosynthesis of Quinol and Quinone parent class, Fatty Acids and Lipid Biosynthesis parent class), which were increased in DS with NcD (File S3).

### 3.5 GM analysis according to *APOE* ε4 allele

Because the *APOE* ε4 allele increases AD risk in both the euploid and trisomic population,^12^ we compared GM between participants with DS who carried (*n =* 10) or not carried (*n =* 48) the ε4 allele. No significant results were found for alpha and beta diversity metrics, whereas beta‐dispersion calculated on weighted Unifrac distances showed significant differences between the two groups (nominal *p*‐value = 0.026, *R^2 ^=* 0.091; File S5). Taxa abundance analysis did not show significant differences at the phylum level, whereas at the family level we identified a reduction of *Bacteroidaceae* (nominal *p*‐value = 0.009) in DS ε4 carriers. At the genus level, *Negativibacillus* was increased in ε4 carriers (nominal *p*‐value = 0.002), whereas *Lachnospiraceae UCG‐010* (nominal *p*‐value = 0.012), *Bacteroides* (nominal *p*‐value = 0.040), and *Lachnoclostridium* (nominal *p*‐value = 0.042) were reduced in ε4 carriers (File S5).

### 3.6 GM analysis according to plasma AD biomarkers

**FIGURE 4:**
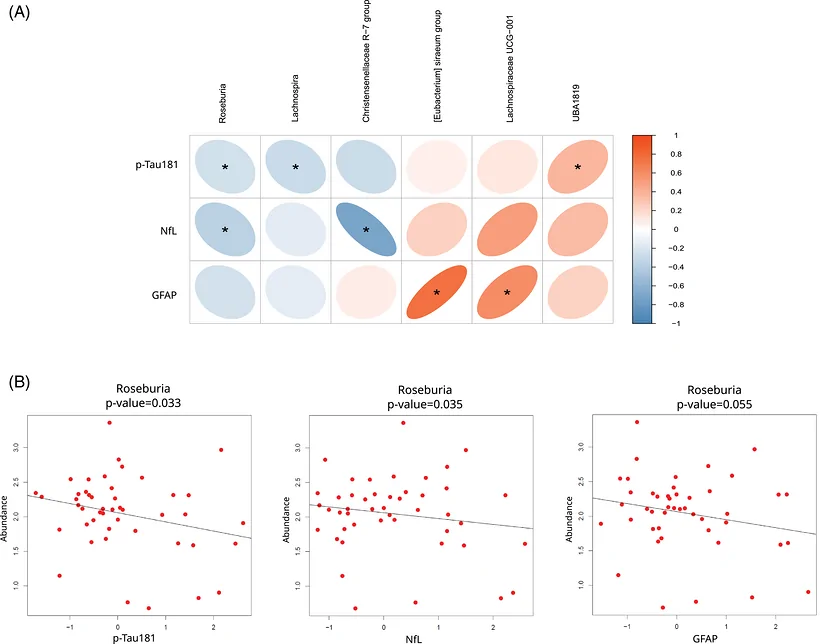
Association analysis of genera abundance and AD plasma biomarkers. A. Correlation matrix between plasma AD biomarkers (p‐tau181, NfL, and GFAP) and genera abundance in DS participants. The matrix reports only the genera that show a significant association with at least one biomarker. Colors indicate Pearson correlation coefficient values. The elliptical shape indicates the strength of correlation between microbial genus and plasma biomarker. In the correlation visualization, bolder colors indicate higher correlations. If the shape of an ellipse bends toward the right, it indicates positive correlation, whereas negative correlation if its shape bending toward the left shows a negative correlation. Sample size *n =* 47. B. Scatterplots reporting an example of the results obtained from the association with AD plasma biomarkers levels. *Roseburia* abundance is represented in association with p‐tau181, NfL, and GFAP plasma levels. Sample size *n =* 46.

Finally, we analyzed the association between GM composition and plasma AD biomarkers in DS participants. No significant associations were found between alpha diversity metrics and plasma levels of p‐tau181, NfL, or GFAP. Conversely, we detected significant associations for specific genera (Figure 4A and File S6). In particular, we found a significant association with p‐tau181 for the thre genera *Roseburia* (nominal *p‐*value = 0.033)*, Lachnospira* (nominal *p‐*value = 0.020), and *UBA1819* (nominal *p‐*value = 0.009); a significant association with NfL for the two genera *Roseburia* (nominal *p‐*value = 0.035) and *Christensenellaceae R‐7 group* (nominal *p‐*value = 0.013); and a significant association with GFAP for the two genera *[Eubacterium] siraeum group* (nominal *p‐*value = 0.005) and *Lachnospiraceae UCG‐001* (*p‐*value = 0.014). Notably, *Roseburia* showed a significant negative association with both p‐tau181 and NfL, and it showed a marginally significant association with GFAP (nominal *p‐*value = 0.055) (Figure 4B and File S6).

As above, the analyses were repeated with the inclusion of anxiolytic or antipsychotic use as covariates. The previously described results were largely confirmed after adjustment for medication use, with most of the associations with AD biomarkers remaining statistically significant or reaching marginal statistical significance (Table S2).

Functional abundance analysis identified no pathway significantly associated with p‐tau181, whereas 55 and 11 pathways emerged as significantly associated with NfL and GFAP, respectively (File S3). In particular, we found several pathways annotated within the Proteinogenic Amino Acid Biosynthesis (4/55), Enzyme Cofactor Biosynthesis (8/55), and Alcohol Degradation (4/55) classes that were negatively associated with NfL. In addition, we identified several pathways annotated within the Fatty Acids and Lipid Biosynthesis (4/55) classes that were positively associated with NfL. Notably, several pathways related to Fermentation to Short‐Chain Fatty Acids class that were significantly reduced in the DS group were also negatively associated with NfL and GFAP plasma levels (File S3).

## 4 DISCUSSION

In this study, we analyzed the gut microbiome composition in adults with DS in comparison with euploid controls, and we further examined how microbiome variation relates to clinical diagnosis of NcD and to the levels of plasma AD biomarkers. To control for potential confounding effects, all the analyses were corrected for sex, age, and, within the DS subgroup, for BMI; the effect of medications on the reported associations was also tested.

Our data suggest no major alterations in the overall GM composition of adults with DS compared to euploid subjects, whereas more fine‐grained differences were observed. In particular, we found that the DS group exhibited weak but significant differences in beta diversity, based on the Bray–Curtis metric; DS also showed a marginally significantly larger dispersion, which is a feature described in several pathological conditions.^45^, ^46^ In our cohort, DS showed no differences in alpha‐diversity metrics or in F/B ratio with respect to CTRL. Conversely, some studies showed more profound alterations in DS microbiota. Differences in alpha diversity with respect to euploid controls were described in children and young adults with DS^28^, ^29^, although with a discordant direction of change in the two available studies. In the study by Cai et al., which included both pediatric and adult participants, differences in beta diversity between DS and CTRL were reported, together with a reduction in the F/B ratio in the DS group^28^. These heterogeneous results can be ascribed to limited sample sizes, variations in experimental and analytical pipelines, and differences in the demographic characteristics of the evaluated cohorts. Accordingly, our results showing no major rearrangements in DS GM are consistent with those reported previously by Biagi et al. in a cohort with geographical origin and demographic characteristics similar to ours.^27^

We did not observe overlap in the results of differential abundance analysis between DS and CTRL groups across the different studies, including ours. Despite the similarities in the demographic characteristics of the cohorts, a direct comparison with Biagi et al. is not possible, as our analytical pipeline filtered out the low prevalence genera reported there as significant (*Parasporobacterium*, *Sutterella*, and *Veillonellaceae*).^27^ Functional abundance analysis in our dataset identified a reduction of pathways related to menaquinone (vitamin K2) biosynthesis in DS. Menaquinone, mainly produced by the GM, is known to improve bone health and prevent coronary calcification, but several studies have also reported a key role in preserving cognitive functions^47^ and brain health.^48^ Animal studies showed that menaquinone administration can reverse cognitive changes induced by antibiotic‐induced gut dysbiosis, pointing to a neuroprotective role mediated by a reduction of oxidative stress and inflammation in intestine and brain.^49^ Therefore, we can hypothesize that the GM changes that we observed in our dataset may contribute to the enhanced neuroinflammation characteristic of DS.^50^

As adults with DS are genetically at higher risk of developing AD at an early age, it is of interest to investigate whether the GM alterations described in euploid AD patients are already present in our entire DS cohort, which includes participants of different ages and with and without cognitive decline. Among the genera identified by differential abundance analysis, *Clostridium sensu stricto 1* and *Intestinibacter* were previously reported to be decreased in patients with AD,^51^, ^52^ whereas they showed the opposite direction of change in our DS cohort. Conversely, *UBA1819* and *Intestinibacter* were reported to be elevated in euploid subjects with a clinical diagnosis of MCI in a Greek cohort,^53^ consistent with our findings. These results suggest that, when considering our entire cohort, the observed GM alterations show some similarities with those reported in MCI, rather than those described in AD patients, suggesting the presence of a potential risk profile. Within this framework, it is worth noting that some studies suggest the presence of stage‐specific GM changes along the AD continuum in the general population, with alterations observed in AD being only partially detected in patients with clinical MCI and vice versa.^23^, ^54^, ^55^

We then specifically evaluated the GM alterations associated with NcD in DS. To the best of our knowledge, only Rosas et al. recently investigated this topic, comparing 14 cognitively stable DS and 6 DS with MCI, defined on the basis of the assessment of subtle cognitive and/or functional decline.^30^ Our dataset included a clinical characterization (diagnosis of major NcD by DSM‐5 criteria) and a biological characterization (plasma biomarkers) of AD‐associated cognitive decline.

When comparing participants with DS with and without NcD, we found a reduction of the *Firmicutes* phylum in the NcD group, in accordance with the results reported by Rosas et al.^30^ The *Firmicutes* phylum includes many short‐chain fatty acid (SCFA) producers with protective and anti‐inflammatory properties,^56^ and its reduction has been reported in AD.^22^, ^57^ Other taxa previously reported altered in AD in the euploid population, like *Proteobacteria* phylum or *Turicibacter* genus,^23^, ^57^ did not show significant differences between DS with and without NcD or fell below the limit of detection in our cohort, respectively. At the same time, our differential abundance analysis revealed other taxa previously reported to be altered in euploid AD patients. Among them, there are the Peptostreptococcaceae family, reduced in the NcD DS group as in AD,^58^ and the *Alistipes* and *Roseburia* genera, respectively, increased and decreased in the NcD group, which have been reported as altered in AD and associated with cognitive decline.^20^, ^23^, ^59^, ^60^, ^61^ In addition, the *Lachnospiraceae UCG‐010* genus, that we found reduced in DS with NcD, was also reported in a study on euploid patients classified as MCI according to neuropsychological evaluation.^62^ Dedicated approaches, such as metabolomic analyzes and experimental model systems, are needed to elucidate the functional consequences of the GM alterations that we observed in DS with NcD. For example, microbial functional gene prediction in our dataset returned an increase in the biosynthesis of ubiquinol‐7, a key component of the electron transport chain in aerobic conditions. This alteration may therefore reflect a shift in microbiota composition toward bacteria that adopt alternative respiratory strategies.^63^

In the general population the strongest genetic risk factor for AD is the genotype of the *APOE* gene, with the ε4 allele conferring a dose‐dependent increase in disease risk.^64^ This association has been replicated also for individuals with DS, where carriers of the ε4 allele exhibit earlier cognitive decline and alterations in positron emission tomography (PET) imaging, CSF, and plasma biomarkers of AD.^12^ So far, only few studies have investigated the impact of *APOE* genotype on gut microbiota in healthy adults and in patients with AD, reporting taxa alterations associated with the ε4 allele.^65^, ^66^, ^67^ In our cohort of adults with DS, ε4 allele carriers had a reduced abundance of the *Bacteroidaceae* family and of the *Bacteroides* genus, consistent with what was reported by Tran et al. in APOE4‐targeted replacement transgenic mice.^67^ It is intriguing that the decrease in *Lachnospiraceae UCG‐010* genus that we observed in DS with NcD characterized also the ε4 carriers in our cohort. The *Lachnospiraceae UCG‐010* genus is a butyrate‐producing bacteria belonging to the family of Lachnospiraceae (like *Lachnoclostridium*, also reduced in DS ε4 carriers). This family tends to have a protective role in the maintenance of brain health, and is reported to be reduced along the AD spectrum.^23^, ^68^

The analysis of the association between taxa abundance and plasma AD biomarkers in participants with DS further expanded the results described above. Notably, the abundance of *Roseburia*—a key butyrate producer with well‐known anti‐inflammatory properties, which we found reduced in DS individuals with NcD—showed negative associations with all plasma AD biomarkers. A recent study on euploid AD patients found that *Roseburia hominis* species was negatively associated with CSF‐based amyloid and p‐tau status.^20^ The same study also identified *Christensenellaceae R7* spp, which was negatively associated with NfL in our dataset and was also reported to be reduced in DS with MCI by Rosas et al.^30^ In addition, the *UBA1819* genus, which we found significantly increased in DS compared to euploid controls, was positively associated with p‐tau181.

In summary, in this study we extended previous findings on GM alterations in adults with DS, reporting some similarities with those observed in euploid individuals with a clinical diagnosis of MCI, possibly indicative of a microbial risk profile. Furthermore, for the first time, we investigated associations between GM and the diagnosis of NcD, as well as AD plasma biomarkers, in individuals with DS. Our results show an overlap with the microbiota changes described in euploid AD patients, supporting the hypothesis that the natural history of AD in DS parallels that of sporadic and autosomal dominant disease.^69^ We acknowledge that our study has several limitations, including the limited sample size and the lack of a detailed dietary assessment. In addition, although sequencing of the V3–V4 region of th 16S rRNA gene is a widely used approach, its resolution is limited at the genus level, restricting taxonomic precision and hindering causal inference. Future studies could benefit from higher‐resolution techniques, such as shotgun metagenomic sequencing. Our results should be also complemented by additional characterizations, like fecal metabolomic profiling, to better elucidate the functional implications of the microbial changes that we observed in the pathogenesis and progression of AD in DS. Of note, inter‐individual variability is particularly relevant in DS, where the onset and progression of dementia follow heterogeneous trajectories, likely owing to complex interactions among genetic, host, and environmental factors, including the gut microbiome. Increasing the cohort size and performing longitudinal assessments are therefore critical to better evaluating the contribution of GM changes to AD in adults with DS. This line of investigation holds substantial promise for future research and may identify novel targets for early intervention against AD in adults with DS.

## CONFLICT OF INTEREST STATEMENT

Camilla Pellegrini, Francesco Ravaioli, Sara De Fanti, Claudia Sala, Magali Rochat, Virginia Pollarini, Barbara Polischi, Alberto Pasti, Margherita Grasso, Maria Rambaldi, Francesco Cardoni, Nicola Grotteschi, Filippo Caraci, Pietro Cortelli, Federica Provini, Raffaele Lodi, Luca Morandi, Piero Parchi, Gian Luca Pirazzoli, Luisa Sambati, Caterina Tonon, and Maria Giulia Bacalini reported no conflicts of interest. Author disclosures are available in the Supporting Information.

## CONSENT STATEMENT

All participants provided their written informed consent for the use of their biological material and clinical data for research purposes. Ethical approval was granted by the local ethics committee of the local health service of Bologna.

## Supporting information



Supporting Information

Supporting Information

Supporting Information

Supporting Information

Supporting Information

Supporting Information

Supporting Information

Supporting Information

Supporting Information

Supporting Information
