# Supplementary material for: Alterations of gut microbiota in Down syndrome and their association with Alzheimer's disease
Source: Alzheimers Dement. 2026 Sep 8;22(9):e71815. doi: 10.1002/alz.71815 (PMC13554992; doi:10.1002/alz.71815)
Supplement: Supplementary file 2 [file ALZ-22-e71815-s005.pdf]

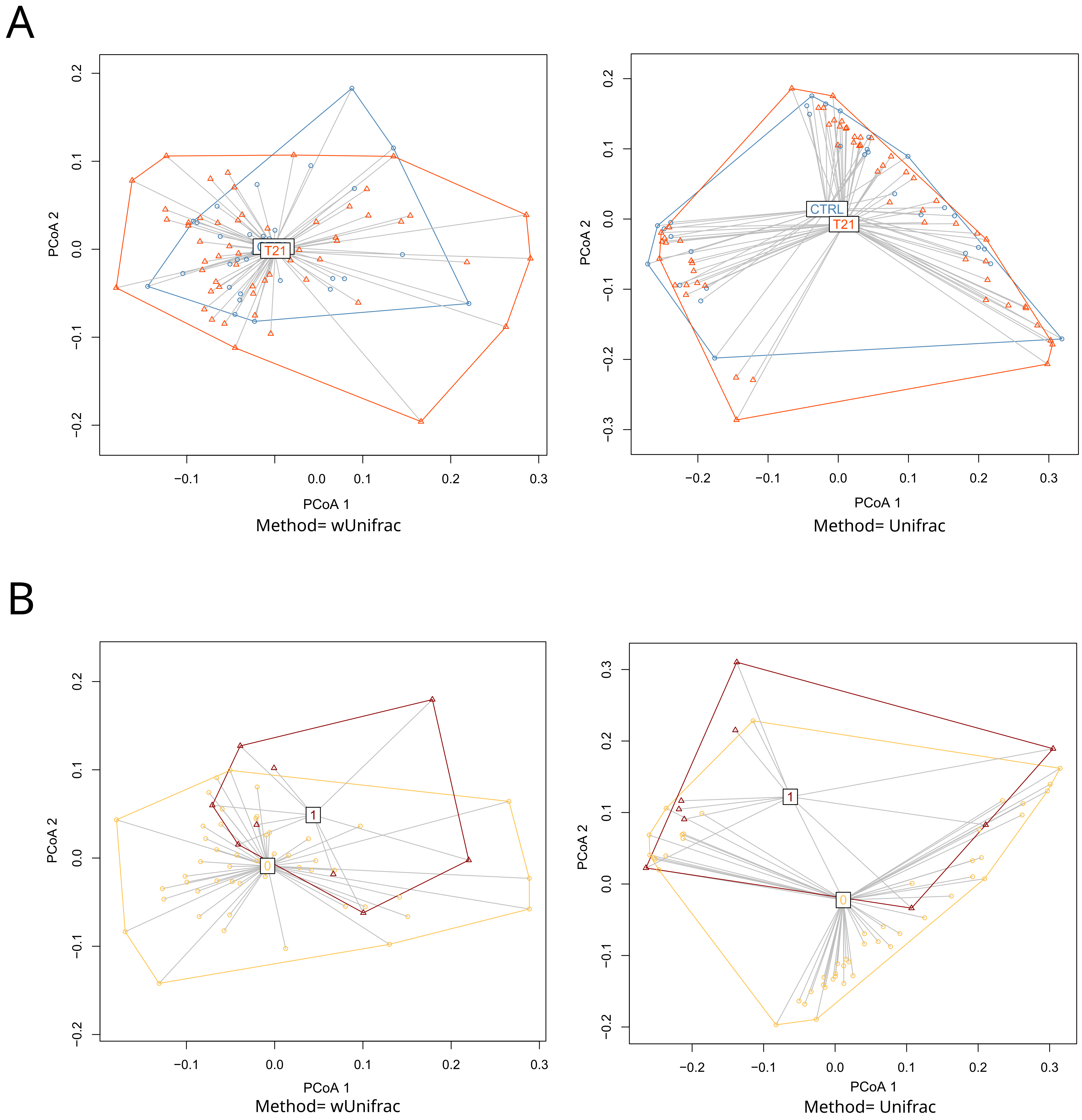

Supplementary Figure 1.
A) PERMDISP beta-dispersion PCoA plot showing separation of DS (red) and CTRL (blue) groups based on weighted (left) and unweighted (right) Unifrac distances. Sample size n=88. B) PERMDISP beta-dispersion PCoA plot showing separation of of DS wNcD (dark red) and DS w/oNcD (yellow) based on weighted (left) and unweighted (right) Unifrac distances. Sample size n=58.
