# Supplementary material for: Alterations of gut microbiota in Down syndrome and their association with Alzheimer's disease
Source: Alzheimers Dement. 2026 Sep 8;22(9):e71815. doi: 10.1002/alz.71815 (PMC13554992; doi:10.1002/alz.71815)
Supplement: Supplementary file 3 [file ALZ-22-e71815-s006.docx]

|  | **Antipsychotics**  p-value Log2FC | | | **Anxiolytics**  p-value Log2FC | | | |  |  |
| --- | --- | --- | --- | --- | --- | --- | --- | --- | --- |
| *DSwNcD vs DSw/oNcD* |  |  |  | | | |  | | |
| Roseburia  Lachnospiraceae UCG-010  Parabacteroides  Alistipes  Butyricicoccus | 0.027*  0.116  0.017*  0.065  0.048* | -1.815  -2.863  2.763  1.514  1.865 | | | 0.014*  0.002*  0.012*  0.565  0.083 | -2.156  -6.553  3.081  0.484  1.740 | | |  |
| *~ p-Tau181*  Roseburia  Lachnospira  UBA1819 | 0.050*  0.008*  0.061 | -0.706  -1.056  0.920 | | | 0.025*  0.010*  0.133 | -0.798  -1.005  0.724 | | |  |
| *~ NfL*  Roseburia  Christensenellaceae R-7 group | 0.050*  0.018* | -1.022  -2.192 | | | 0.037  0.017* | -1.099  -2.209 | | |  |
| *~ GFAP*  [Eubacterium] siraeum group  Lachnospiraceae UCG-001 | 0.005*  0.015* | 2.434  1.948 | | | 0.003*  0.028* | 2.528  1.773 | | |  |

**Supplementary Table 2. Genus-level analysis including medication use as covariates**

The genera listed were significantly different between the DSwNcD and DSw/oNcD groups and showed significant associations with the biomarkers p-Tau181, NfL, and GFAP. *, p-value<0.005 calculated by DESeq2 analysis

Abbreviations: (DS, Down Syndrome participants; DSw/oNcD, Down Syndrome participants with Neurocognitive Disorder; DSwNcD, Down Syndrome participants without Neurocognitive Disorder subjects; *Log2FC*, Log2 Fold Change; *p-value*, nominal p-value)
