# Supplementary material for: Alterations of gut microbiota in Down syndrome and their association with Alzheimer's disease
Source: Alzheimers Dement. 2026 Sep 8;22(9):e71815. doi: 10.1002/alz.71815 (PMC13554992; doi:10.1002/alz.71815)
Supplement: Supplementary file 4 [file ALZ-22-e71815-s008.docx]

|  | **DS**  **(n=58)** | ***DSw/oNcD***  ***(n=49)*** | ***DSwNcD***  ***(n=9)*** | ***p*** |
| --- | --- | --- | --- | --- |
| *Medications (n, percentage)* |  |  |  |  |
| Thyroid drugs | 31 (53%) | 25 (51%) | 6 (67%) | 0.616 |
| PPI | 7 (12%) | 4 (8%) | 3 (33%) | 0.115 |
| Antidepressants | 13 (22%) | 11 (22%) | 2 (22%) | 1 |
| Trazodone | 4 (7%) | 2 (5%) | 2 (22%) | 0.208 |
| Anxiolytics | 5 (9%) | 1 (2%) | 4 (44%) | <0.001* |
| Antipsychotics | 16 (28%) | 10 (20%) | 6 (67%) | 0.014* |
| Mood stabilizers | 6 (10%) | 3 (6%) | 3 (33%) | 0.061 |
| Anti gout | 13 (22%) | 9 (18%) | 4 (44%) | 0.19 |
| Melatonina | 7 (12%) | 4 (8%) | 3 (33%) | 0.115 |
| *Nutritional supplements (n)* |  |  |  |  |
| Folic Acid | 34 (59%) | 29 (59%) | 5 (56%) | 1 |
| Iron | 6 (10%) | 6 (12%) | 0 (0%) | 0.608 |
| Vitamin D | 39 (67%) | 33 (67%) | 6 (67%) | 1 |
| Vitamin B | 20 (34%) | 15 (31%) | 5 (56%) | 0.287 |

**Supplemenray Table 1. Reported medication and supplement intake in DS group**

Abbreviations: *DS*, Down Syndrome participants; *DSw/oNcD*, Down Syndrome participants with Neurocognitive Disorder; *DSwNcD*, Down Syndrome participants without Neurocognitive Disorder subjects; PPI, Proton Pump Inhibitors; *p*, p-value. All the comparison were performed using chi-square χ^2^ test

*, significant
